# Sparse Recovery Optimization in Wireless Sensor Networks with a Sub-Nyquist Sampling Rate

**DOI:** 10.3390/s150716654

**Published:** 2015-07-10

**Authors:** Davide Brunelli, Carlo Caione

**Affiliations:** 1 University of Trento, Via Sommarive 9, Trento 1-38122, Italy; 2 University of Bologna, Viale Risorgimento 2, Bologna 1-40136, Italy; E-Mail: carlo.caione@unibo.it

**Keywords:** compressed sensing, wireless sensor networks, distributed compressed sensing, embedded software, low-power electronics

## Abstract

Compressive sensing (CS) is a new technology in digital signal processing capable of high-resolution capture of physical signals from few measurements, which promises impressive improvements in the field of wireless sensor networks (WSNs). In this work, we extensively investigate the effectiveness of compressive sensing (CS) when real COTSresource-constrained sensor nodes are used for compression, evaluating how the different parameters can affect the energy consumption and the lifetime of the device. Using data from a real dataset, we compare an implementation of CS using dense encoding matrices, where samples are gathered at a Nyquist rate, with the reconstruction of signals sampled at a sub-Nyquist rate. The quality of recovery is addressed, and several algorithms are used for reconstruction exploiting the intra- and inter-signal correlation structures. We finally define an optimal under-sampling ratio and reconstruction algorithm capable of achieving the best reconstruction at the minimum energy spent for the compression. The results are verified against a set of different kinds of sensors on several nodes used for environmental monitoring.

## 1. Introduction

The recent evolution of sensing devices and the availability of new solutions and techniques in the area of WSN have increased the expectation of WSN applications. Today, WSN is struggling with issues concerning the battery lifetime and power consumption. Recent research advances in the field of data compression have triggered the possibility to exploit the minimization of storage and communication payload, with the goal to extend as much as possible the lifetime of the nodes.

Recently, compression algorithms have gained a lot of interest, in particular the ones capable of exploiting the fact that the majority of the signals of interest in WSN applications has sparse representation in terms of some basis [1-3]. Compressed sensing (CS) has been used as a new approach to simultaneous sensing and compressing, fostering a potentially large reduction in sampling and computational costs.

CS builds on the works [4-6], which demonstrated that if a signal can be compressed using classical transform coding techniques and its representation is sparse in some basis, then a small number of projections on random vectors contain enough information for approximate reconstruction. The compression comes from the fact that the number of these measurements is usually smaller than the number of samples needed if the signal is sampled at the Nyquist frequency. In general, if a signal has a sparse representation in one basis, it can be recovered from a small set of measurements onto a second measurement basis that is incoherent with the first.

While a rich literature has been developed about the mathematical aspects of CS and the reconstruction algorithms used to perform reconstruction (*i.e.*, [7,8], relatively limited attention has been paid to practical implementation of CS on resource-constrained nodes, such as those typically used in WSN deployments. Distributed CS (DCS) [9,10] is probably the most prominent framework dealing with the sparsity and compressibility of signal ensembles tailored on distributed sensor nodes where signals are each individually sparse in some basis, but a correlation from sensor to sensor does exist.

Moreover, the great majority of the papers addressing CS and DCS deals with a purely digital implementation of CS, which consists of sampling the signal at a given frequency (e.g., Nyquist or above) and then compressed using CS with dense encoding matrices. Nevertheless, when natural signals have a relatively low information content, as measured by the sparsity of their spectrum, the theory of CS suggests that randomized low-rate sampling may provide an efficient alternative to high-rate uniform sampling. This technique is usually referred to as analog CS, and it is a novel strategy to sample and process sparse signals at a sub-Nyquist rate [11].

In this paper, we address the problem of energy consumption for sensor nodes performing CS and DCS when both digital and analog CS are considered. Our contribution is: (i) to establish a common energy framework in which a fair comparison can be made by modeling the nodes when real signals are considered for reconstruction and real resource-constrained hardware is used to perform the compression; (ii) to investigate the impact of CS parameters for compression on nodes' lifetime; this was only partially discussed in [12]; (iii) to investigate if low-rate CS (CS with sub-Nyquist sampling) can be exploited to reconstruct environmental signals with good quality; and (iv) to propose design parameters for low-rate CS that are able to achieve a superior reconstruction quality with the minimum energy expenditure, so as to prolong the lifetime of the whole network.

The rest of this paper is organized as follows. Section 2 surveys related works. Section 3 gives a brief introduction about compressive sensing background. In Section 4, the energy consumption modeling for CS is addressed when small COTSWSN nodes are used. The low-rate CS is proposed in Section 5, and the reconstruction analysis is presented in Section 6. In Section 7, we discuss the conclusions.

## 2. Related Works

The problem of data gathering and compression using CS is widely developed in the literature. Even though a lot of attention is paid to reconstruction algorithms and mathematical aspects, practical aspects and implementation problems have been gaining a lot of interest lately.

The general problem of using CS in WSNs is investigated in several works, like in [13], where the authors analyze synthetic and real signals against several common transformations to evaluate the reconstruction performance, or in [14], where the measurement matrix is created jointly with routing, trying to preserve a good reconstruction quality. Furthermore, in [15], the authors improve reconstruction by reordering input data to achieve a better compressibility. In general, all of these papers address the problem of the signal reconstruction, but they lack a real consideration about energy involved in compression. When real hardware is considered, considerations about CS have to be revised.

One of the first papers trying to address the problem of energy consumption for compression dealing with the problem to generate a good measurement matrix using as low energy as possible is [3]. In this work, the research is focused on wireless body sensor networks (WBAN) for real-time energy-efficient ECG compression. Other works that focus on bio-signals and WBANs are [16,17]. This is a quite different research field with respect to WSNs, where the presence on several nodes sensing the same environment permits one to exploit the distributed nature of the signals to improve the quality of recovery. However, CS is today applied in several other signal processing fields, from video compression [2] to underwater acoustic OFDMtransmission [18] and to air quality monitoring [19].

In fact, several works, like [20] or [21], deal with the use of CS when multiple nodes are used in a joint reconstruction. The best known technique used to exploit the existing correlation among several nodes in a WSN is distributed compressed sensing (DCS) [9,10], which permits new distributed coding algorithms for multi-signal ensembles that exploit both intra- and inter-signal correlation structures.

Besides the classical digital implementation of CS used in all of the aforementioned papers, in this paper, we deal also with CS when the signals are sampled at a sub-Nyquist frequency. Usually, in the literature, this compression technique is referred to with the name of analog CS. This is because, usually, the subsampling is performed at the ADClevel, dropping samples during the acquisition and analog-to-digital conversion stage. For example, in [22], the effects of circuit imperfections in the analog compressive sensing architectures are discussed.

While it is common in the literature to find papers like the two aforementioned addressing the problem of analog CS with a focus on the hardware called analog-to-information converters (AICs), other works investigate the problem from a higher system-level prospective when the samples are not discarded by the ADC architecture, but by the device performing the sensing. One of the papers dealing with this specific case is [23], where the analysis on energy consumption is totally neglected, and it is strictly related to the specific application of the pulse oximeter. Differently from environmental signals, the signals obtained by the oximeter present a much higher temporal correlation, presenting small variations in its temporal evolution.

Furthermore, in [24], the authors use a sparse generated matrix adjusting the sampling rate to maintain an acceptable reconstruction performance while minimizing the energy consumption. In this work, the authors use the reconstruction quality to give the node a feedback that is used to modify the sampling pattern. Differently from this work, the authors do not address the problem of investigating different reconstruction algorithms, and they just rely on simple BPDN or LASSOfor reconstruction. Neither to they try to exploit potential correlations among signals and nodes or training to increase the quality of the recovered signal.

Even in [25], the usage of sparse measurement matrices is investigated, and even though the energy consumption in a WSN is taken into consideration, in the paper, there is no precise analysis on the energy for compression nor a real trade-off between power consumption and reconstruction quality, like we do in our work.

In [26], the authors use a weighted form of the basis pursuit to reconstruct signals gathered using a sparse measurement matrix addressing also the problem of the energy spent in generating the random projection matrix on the node itself. Nevertheless, the aim of the paper is quite different from ours: the authors in [26] want to detect a specific event characterized by a well-defined frequency, and this makes it easier to train the reconstruction algorithm to detect the specified event; whereas in our approach, we address the reconstruction without any priorsabout the signal to recover and using temporal or spatial correlation as data training for reconstruction.

Related to this work is also [27], where a sparse matrix is generated considering the energy profile of the node and, even considering a set of environmental signals similar to those ones reported in this paper, the authors do not try to exploit the inter-signals correlation properties. While in [28], the authors introduce the random access compressed sensing, a form of low-rate CS, but their focus is on the network architecture investigating the network design more than using compressive sensing for data compression.

## 3. CS and DCS: A Mathematical Background

For a band-limited signal *x*(*t*) of duration *T*, let *x*(*n*), 1 ≤ *n* ≤ *N* be its discrete version. The Nyquist sampling theorem states that in order to perfectly capture the information of the continuous signal *x*(*t*) with band-limit *B*_nyq_/2 Hz, we must sample the signal at its Nyquist rate of *B*_nyq_ samples/s. Thus:

(1) $$x\left(n\right)={x\left(t\right)|}_{t={nT}_{s}}$$

such that *T_s_* ≤ 1/*B*_nyq_ and *NT_s_* ≤ *T*. Sampled in time, the signal that we want to acquire is represented by an *N*-dimensional vector of real numbers **x**.

In the standard CS setting, one is concerned with recovering this finite-dimensional vector **x** ∈ ℝ*^N^* from a limited number of measurements. A typical assumption is that the vector x is sparse. The sparsity of a signal is usually indicated as the ℓ_0_-norm of the signal, where the ℓ*_p_*-norm ‖ · ‖*_p_* is defined as:

(2) $${\left\|\alpha \right\|}_{p}={\left(\sum_{i=0}^{N-1}{\left|\alpha_{i}\right|}^{p}\right)}^{1/p}$$

with ***α*** ∈ ℝ*^N^*. Thus, if the signal **x** is sparse, this means that there exists some *N* × *N* basis or dictionary **ψ** ∈ ℝ*^N^*^×^*^N^*, such that, for any substance of **x** there is an *N*-dimensional vector **α**, such that **x** = **ψα** and ‖**α**‖_0_ ≤ *K* with *K* ≪ *N*.

CS theory demonstrates that this kind of signal can be compressed using a second different matrix **Φ** ∈ ℝ*^M^*^×^*^N^* with *M* ≪ *N*. The compression procedure can be written as **y** = **Φx**, where **y** is the *M*-dimensional measurements vector.

Since **ψ** is usually defined by the signals characteristic and it is considered fixed, one seeks to design **Φ**, so that *M* is much smaller than *N*.

Having the measurements vector **y**, the recovery of the original signal **x** can be obtained by the inversion of the problem:

(3) y=Θα=ΦΨα

In general, this is not an easy task, since the matrix **Θ** ∈ ℝ*^M^*^×^*^N^* is rectangular with *M* ≪ *N*. Fortunately, the fact that **x** is sparse relaxes the problem a bit, opening the way to the use of optimization-based reconstruction or iterative support-guessing reconstruction.

The most common optimization-based method is the so-called basis pursuit (BP) method that looks for the “most sparse” solution for which the ‖**α**‖_1_ is minimum. In formulas:

(4) $$\hat{\alpha }=\arg\min\;{\left\|\alpha \right\|}_{1}\;\text{s}.\text{t}.\;\text{y}=\Theta \alpha =\Phi \Psi \alpha$$

CS proves that if the two matrices **Φ** and **ψ** are incoherent (elements of the matrix **Φ** are not sparsely represented in the basis **ψ**) and the original signal **x** is compressible, then we can recover ***a*** with high probability [29].

In case the sensors, which produce data, are close each other (as is usual in a WSN), the signals can be assumed similar and the outputs correlated. We can then expect that the ensemble of these signals has an underlying joint structure (inter- and intra-correlation) so that it is possible to exploit to further compress data.

In an ensemble of *J* signals, we can denote with **x***_j_* ∈ ℝ*^N^* the *j*-th signal with *j* ∈ {1,2,…, *J*}. As done before for the single signal CS, for each signal **x***_j_* in the ensemble, we can have a sparsifying basis **ψ** ∈ ℝ*^N^*^×^*^N^* and a measurement matrix **Φ***_j_* ∈ ℝ*^MJ^*^×^*^N^*, such that **y***_j_* = **Φ***_j_***x***_j_* with *M_j_* ≪ *N* and **x***_j_* = **ψ*α****_j_*. Even though the DCS theory proposes three different models [9,10] for jointly-sparse signals, it is possible to consider JSM-2 as the most suitable model to describe the ensemble of signals, as those ones are typically gathered by nodes in a WSN.

In the JSM-2 model, all signals share the same spare set of basis vectors, but with different coefficients. If ***α****_j_* ∈ ℝ*^N^* is the coefficients vector for the basis **ψ**, which is not zero only on a common set **Ω** ∈ {1, 2,…, *N*} of coefficients, we have |**Ω**| = *K* with **Ω** being the same for all the signals. The reconstruction can be performed via greedy algorithms, such as simultaneous orthogonal matching pursuit (SOMP) or the more promising DCS-SOMP [30].

## 4. Compressive Sensing in Embedded Systems

### 4.1. Hardware and Compression

In this subsection, we want to analyze the real potential of CS aiming at low-complexity energy-efficient data compression on resource-constrained WSN platforms.

CS is usually considered as a suitable approach for data acquisition and compression in WSNs. It is claimed in [31] to be particularly attractive for energy-constrained devices for at least two reasons: (1) the compression is agnostic of the specific properties of the signal and is performed through a small number of linear independent measurements; and (2) the small number of measurements can be transmitted to a remote gathering center where they can be accurately reconstructed using complex, nonlinear and energy expensive decoders [12].

Nevertheless, the energy spent in compression is often underestimated in the literature. When implemented in software, data compression goes through several matrix-vector multiplications, as seen in Section 3, that are not negligible, especially when resource-constrained nodes are used for compression and for the generation of the measurement matrix.

The hardware used as a reference in our tests is a wireless node by ST microelectronics, the STM32W108, which is a fully-integrated SoC with a 2.4-GHz IEEE 802.15.4-compliant transceiver, 32-bit 24-MHz ARM Cortex-M3 microprocessor, 128-KB Flash and eight-Kbyte of RAM memory. Two additional sensors, Sensirion SHT21, are considered on the board. The microcontroller has no floating point unit, and it uses software emulation to overcome this limitation. The compiler used for compiling benchmarks is Sourcery CodeBench Lite Edition, and the code is compiled with -O3optimization. The time measurement is performed using the debug registers in the ARM core capable of accurately measuring the number of cycles spent in performing a certain operation. Data for power consumption of the various subsystems are not reported for lack of space. For reference, the reader can refer to the datasheets of microcontroller [32] and sensors [33]. Our tests and simulations track the reported datasheet values with high fidelity.

Compression using CS can be performed using different kinds of compression matrices **Φ**. In the literature, it is possible to find a plethora of papers arguing on different kind of sensing matrices [34]. As seen in Section 3, the only requirement is that the sensing matrix is highly incoherent with the sparsifying basis **ψ**. Such a property is practically verified for random matrices, such as random matrices with independent identically distributed (i.i.d.) entries. Interestingly, many efficient sensing matrices can be generated having different characteristics and, hence, different memory and power footprints; moreover, they require a different number of bytes for encoding and then storing.

**Figure 1:**
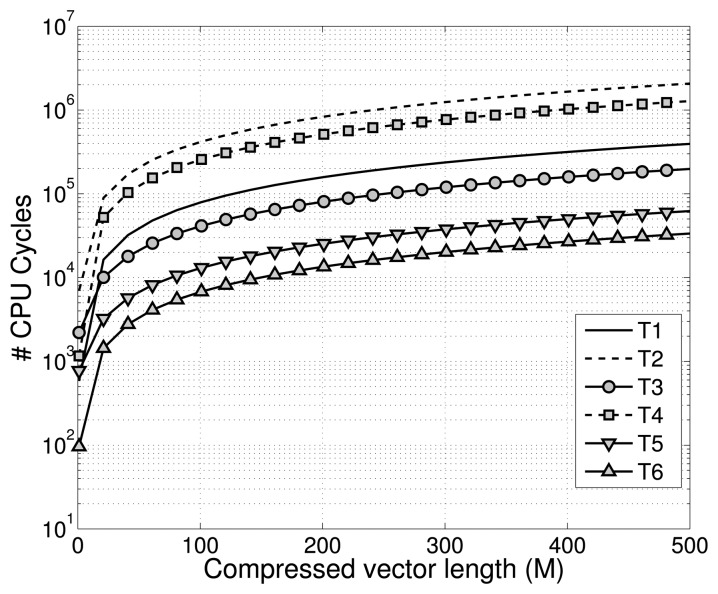
Number of CPU cycles required to compress a sample using different random **Φ** matrices varying the compression factor. (T1) Matrix with random 16-bit fixed-point values. (T2) Gaussian matrix generated using a Box-Muller transformation with mean zero and variance 1/*M*. (T3) Matrix with random floating point values. (T4) Same as T2, but the matrix is generated with the Ziggurat method. (T5) Entries of the matrix are generated from the symmetric Bernoulli distribution with $P\left(\Phi_{jk}=\pm 1\sqrt{M}\right)=1/2$. (T6) Same as T5 with *P*(Φ*_jk_* = ±1) = 1/2.

In Figure 1, the number of cycles required by a microcontroller to generate the compression matrix and to perform the compression of a single sample for different kinds of measurement matrices is shown. The differences are mainly due to: (1) the computational workload required for generating the random vectors for the compression, since in some cases, the generation implies the use of complex and computationally-intensive functions, such as *sqrt* or *log*; and (2) the time spent in multiplication of the vector against the sample that, especially in the case of floating point numbers, is not negligible.

### 4.2. Power Consumption Model

When CS is used to perform compression in a WSN, the type of compression matrix strongly affects also the power consumption of other subsystems: (1) the longer the time necessary to compress the data, the longer the node has to be awake before switching back to sleep mode to save energy; (2) the number of bytes required to encode the compression matrix is not the same for all of the matrices **Φ**; (3) following from the previous point, the time and space required by the micro-controller to store data in non-volatile memory is different; and (4) the energy spent in transmission is different for measurement matrices.

To evaluate the influence of the choice of measurement matrix and other compression parameters, in this subsection, we introduce an architecture-level power consumption model to evaluate the power consumption of the nodes when compression is performed using different parameters for compression, and we compare the results against the power spent to transmit data without any kind of compression. Using this power model and feeding it with data coming from real hardware, we can easily evaluate how changing the parameters influences the energy consumption of the system, enabling design space exploration.

The hardware taken as the reference (already described in this section) is an STM32W108 node acquiring data from the two on-board sensors. The network is organized as a star, a very common topology for practical WSN deployments [35].

During the simulation involving no compression, the node wakes up, samples data from the two sensors and sends them out to a collector center. Afterwards, it goes back to sleep mode waiting for the next cycle. The energy spent in each cycle can be written as:

(5) $$E=E_{\text{sleep}}+E_{\text{setup}}+E_{\text{sample}}+E_{\text{trans}}$$

where *E*_sleep_ is the energy spent in sleep mode, *E*_setup_ is the energy used for waking up and setting up the device, *E*_Sample_ is the energy for sampling each sensors and *E*_send_ is the energy used to send the acquired data. Expanding each term, we have:

(6) $$\begin{array}{ll} E= & T_{\text{sleep}}\cdot \left(P_{\text{sleep}}+P_{\text{soff}}+P_{\text{toff}}\right)+\\ & T_{\text{setup}}\cdot \left(P_{\text{mcu}}+P_{\text{soff}}+P_{\text{toff}}\right)+\\ & T_{\text{sample}}\cdot \left(P_{\text{sample}}+P_{\text{sactive}}+P_{\text{toff}}\right)\\ & T_{\text{trans}}\cdot \left(P_{\text{comm}}+P_{\text{soff}}+P_{\text{trans}}\right) \end{array}$$

where *T*_sleep_, *T*_setup_, *T*_sample_, *T*_trans_ are the duration of each respective phase. *P*_sleep_ is the power consumed in sleep mode; *P*_soff_ is the power absorbed from sensors when sleeping; *P*_toff_ is the power consumption of the transceiver when the node is in sleep mode. *P*_mcu_ is the power consumed by the MCU; *P*_sample_ is the power spent for data acquisition; *P*_sactive_ is the power consumed by sensors; *P*_comm_ is the power consumption for filling the transceiver output buffer; and finally, *P*_trans_ is the power for sending data. All of the values for the power consumption or timing are actually measured on the hardware.

When CS is used to compress data, the compression is performed after the node has acquired *N*_acc_ samples. Thus, the energy consumption in each cycle is:

(7) $$E_{\text{CS}}=\left(N_{\text{acc}}\cdot \left(E_{\text{sleep}}+E_{\text{setup}}+E_{\text{sample}}+E_{\text{store}}\right)+E_{\text{nv}}+E_{\text{comp}}+E_{\text{trans}}\right)/N_{\text{acc}}$$

where *E*_store_ is the energy to store the acquired sample in non-volatile memory, *E*_nv_ is the energy spent during the recovery of the data from non-volatile memory and *E*_comp_ is the energy for compression. In detail:

(8) $$\begin{array}{ll} E_{\text{CS}}= & (N_{\text{acc}}\cdot (T_{\text{sleep}}\cdot \left(P_{\text{sleep}}+P_{\text{soff}}+P_{\text{toff}}\right)+\\ & T_{\text{setup}}\cdot \left(P_{\text{mcu}}+P_{\text{soff}}+P_{\text{toff}}\right)+\\ & T_{\text{sample}}\cdot \left(P_{\text{sample}}+P_{\text{sactive}}+P_{\text{toff}}\right)+\\ & T_{\text{nv}}\cdot \left(P_{\text{store}}+P_{\text{soff}}+P_{\text{toff}}\right)+\\ & T_{\text{store}}\cdot \left(P_{\text{soff}}+P_{\text{toff}}+P_{\text{store}}\right)+\\ & T_{\text{comp}}\cdot \left(P_{\text{soff}}+P_{\text{toff}}+P_{\text{comp}}\right)+\\ & T_{\text{trans}}\cdot \left(P_{\text{comm}}+P_{\text{soff}}+P_{\text{trans}}\right))/N_{\text{acc}} \end{array}$$

with self-explanatory meaning of the symbols.

**Figure 2:**
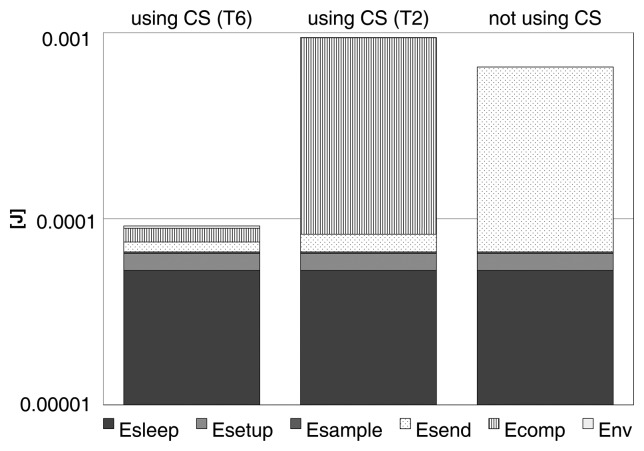
Energy spent in one sampling cycle when CS is used to compress the sample compared to the energy consumed when the sample is sent without compression. The first bar refers to CS when the measurement matrix is obtained from a Bernoulli distribution (T6), while for the second bar, the compression is performed using a Gaussian matrix (T2) (simulation parameters: *N*_acc_ = 512, *M* = 100, *T*_sleep_ = 10 s).

In Figure 2, the result of simulations is reported when *N*_acc_ = 512, *M* = 100, *T*_sleep_ = 10 s with an overhead of 10 bytes for each packet sent. The other parameters in Equations (6) and (8) are derived from these values and the hardware specification data in the datasheets. The two compression matrices used in the simulation when CS is performed are: (T2) Gaussian matrix generated using a Box-Muller transformation with mean zero and variance 1/*M* and (T6) the matrix generated from the symmetric Bernoulli distribution *P*(Φ*_jk_* = ±1) = 1/2. According to Figure 1 these two matrices define the energy consumption boundary for CS.

The result of the simulation clearly shows how compressing data with CS does not always determine an actual savings in power consumption. For all of the cases, the energy spent in sleep mode, the energy for sampling and the energy for setting up the node after sleep are obviously the same. The differences are related to the energy for compression and for sending the data.

Using a complex compression matrix (T2) is really expensive in terms of energy consumption; thus, the overall power consumption is higher with CS than without any compression. Differently, when a simpler matrix is used (T6) the energy for compression becomes negligible, and the power consumption abruptly decreases. A huge difference between using CS and not using compression is also in the power for sending data due to two different factors: (1) the number of bytes sent; and (2) a better packetization, since the compressed vector is sent at the end of the *N*_acc_ cycles, permitting one to maximize the number of compressed samples that fit in the packet payload [36].

## 5. Low-Rate Compressive Sensing

In this section, we want to investigate how it is possible to further reduce the energy consumption by means of simpler sparse measurement matrices and acting on the number of samples gathered by the node.

In classical acquisition systems (as in the digital CS seen before), samples are taken regularly on the time axis at a given rate (usually not less than the Nyquist one). A particular form of CS, called analog CS, relies on random sampling to avoid this regularity and aims to produce a number of measurements that, on average, are less than those produced by Nyquist sampling, while still allowing the reconstruction of the whole signal thanks to sparsity and other priors.

While usually analog CS is performed by means of specialized hardware encoders, we want to study whether analog CS is a suitable technique to be performed on WSNs nodes and whether this peculiar form of compression, which we call low-rate CS (LR-CS), is still able to reconstruct the original signals of interest with satisfying quality.

From a mathematical point of view, the problem is still the same as seen in Equation (3), which is different in the form of the measurement matrix **Φ**. Let B denote an *M*-dimensional vector, each element of which contains a unique entry chosen randomly between one and *N*. In analog CS, the measurement matrix **Φ** is a sparse *M* × *N* matrix, where the *i*-th row of the matrix is an all-zero vector with one at the location given by the *i*-th element of B. This is a very simple measurement matrix, energetically inexpensive to generate and store and permits also savings on the number of samples to gather.

Practically, using this kind of measurement matrix means that the node is required only to randomly gather *M* samples with an under-sampling ratio of order *ρ* = *M*/*N*. As done before, the energy consumption on average after the *N*_acc_ sampling period is:

(9) $$E_{\text{sub}}=\left(M\cdot \left(E_{\text{setup}}+E_{\text{sampl}}+E_{\text{store}}\right)+N_{\text{acc}}\cdot E_{\text{sleep}}+E_{\text{nv}}+E_{\text{send}}\right)/N_{\text{acc}}$$

**Figure 3:**
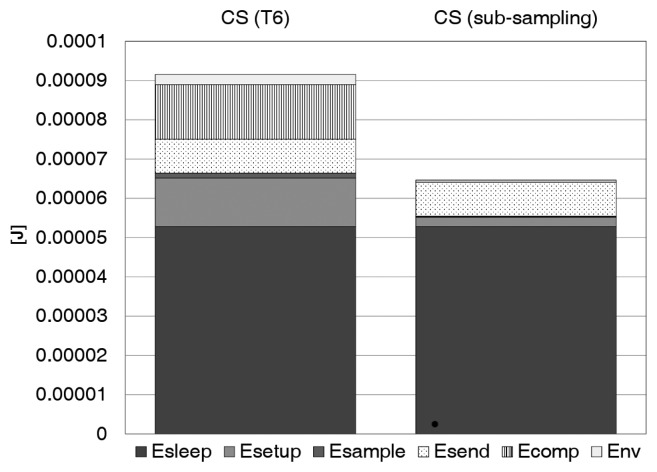
Energy comparison between digital and low-rate compressive sensing (CS) (simulation parameters: *N*_acc_ = 512, *M* = 100, *T*_sleep_ = 10 s).

In Figure 3, the comparison between digital and low-rate CS is reported. As inferred from Equations (7) and (9), the energy savings is mainly due to three factors: (1) there is no energy spent in compression for the analog version of CS; (2) the contribution of *E*_setup_, *E*_sampl_ and *E*_store_ is reduced by a factor *ρ*; and (3) *E*_nv_ is decreased since the number of bytes to store in flash is reduced.

**Figure 4:**
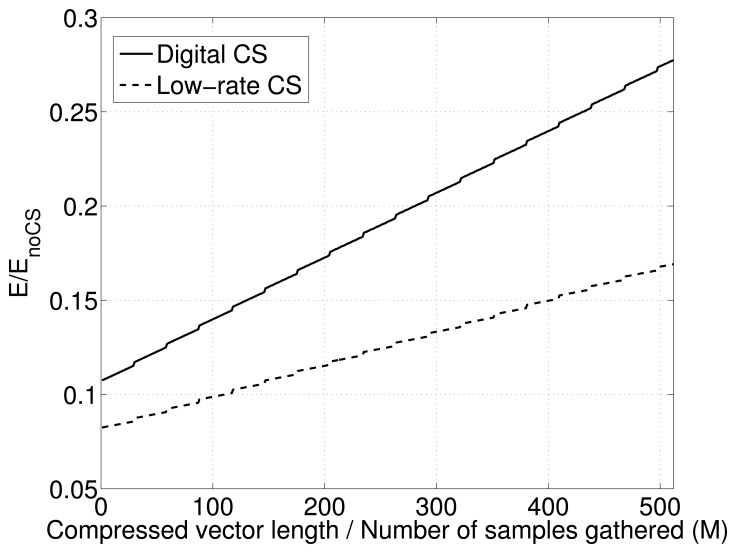
Energy comparison between digital and low-rate CS varying the compressed vector size for digital CS and the number of samples gathered for the low-rate CS.

In Figure 4, the comparison between the energy spent for low-rate and digital CS is reported, normalizing the energy with respect to the energy spent when no compression is applied. The low-rate CS is always more convenient with respect to the digital CS. In the plot is also visible the influence of the packet overhead on the power consumption that creates small abrupt increases in energy consumption when an additional packet has to be sent.

Having verified that using low-rate CS and a sparse measurement matrix, the node can save energy, the problem shifts to verify whether low-rate CS can be used in practice to reconstruct signals gathered by WSNs nodes deployed in a real environment.

## 6. WSN Data Reconstruction for Low-Rate CS

In this section, we want to investigate the performance of several reconstruction algorithms to check if there is an algorithm that is better able than others to cope with low-rate CS and that can guarantee a good signal recovery. Moreover, we want to address the problem of choosing a suitable sampling pattern for the low-rate CS, since the sampling pattern chosen is strictly related to the quality of the recovered signal during the reconstruction phase.

**Figure 5:**
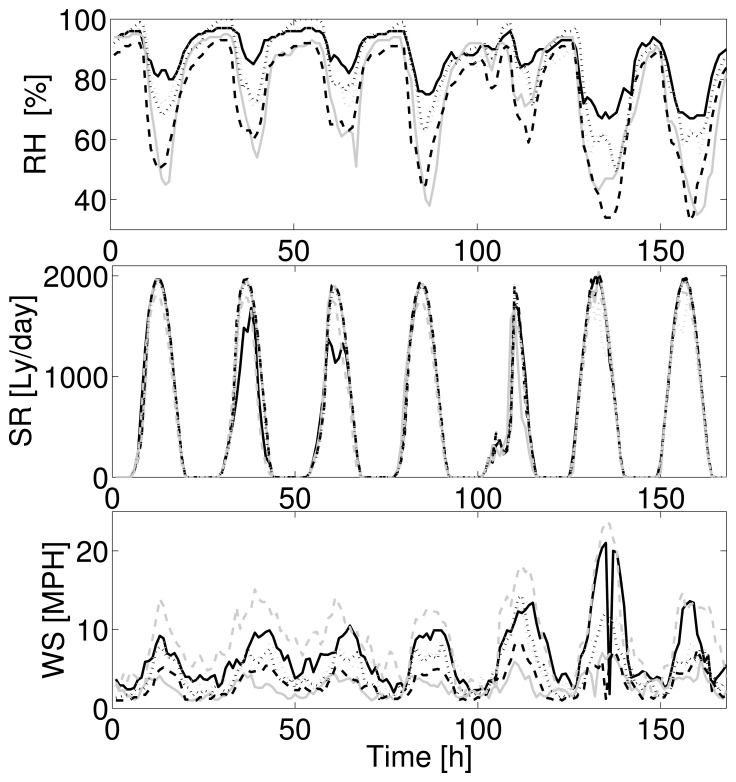
Signals ensembles for (**a**) relative humidity (RH), (**b**) solar radiation (SR) and (**c**) wind speed (WS) for seven different weather stations near Monterey (CA). Each different line in each sensor plot refers to a different node: each kind of sensor presents a different level of correlation among different nodes.

In our experiments, we consider data coming from the CIMIS [37] dataset that manages a network of over 120 automated weather stations in the state of California. We take as the reference the data collected during the 23rd week of 2012 by seven different weather stations near Monterey (CA). For our simulations, we refer to three different kinds of sensors: temperature, relative humidity and wind speed, as reported in Figure 5. The ensemble of signals is chosen, such that it includes periodic and highly correlated signals (temperature and relative humidity) with less correlated signals (wind speed).

In our model, the seven nodes are deployed in the same IEEE 802.15.4 star network. The power consumption for each node adheres to the same model as described in Section 4. In each simulation cycle, each node samples the signal for a certain period, called the acquisition period, collecting a certain number of samples before compressing these samples and sending out the compressed vector toward a central collector. The acquisition period is supposed to be the same for each node, and each node uses the low-rate CS for compressing data. The sparse compression matrix **Φ** used for compression is locally generated by each node using its own ID and the timestamp as the seed for generation. The compressed vectors are gathered by the central coordinator, and here, the original signals are recovered using different algorithms.

Two different sampling patterns for the generation of the measurement matrix **Φ** are considered in this section: (1) uniform sampling (US) pattern; and (2) non-uniform sampling (NUS) pattern. In the uniform sampling pattern, the inter-measurement intervals are constant Δ*k_j_* = *k_j_*_+1_ − *k_j_* = Δ*k* = *γ*Δ*k_min_* where Δ*k_min_* is the minimum sampling period of the ADC and *γ* = ┌*N*/*M*┐, whereas in the non-uniform sampling pattern, the inter-sample period is randomly chosen between [Δ*k_min_*, ∞].

We carry the reconstruction using several algorithms, distributed and non-distributed and evaluate the quality of reconstruction using the SNR expressed in dB:

(10) $${\text{SNR}}_{\text{dB}}=20\cdot \log_{10}\frac{{\left\|\text{x}\right\|}_{2}}{{\left\|\text{x}-\hat{\text{x}}\right\|}_{2}}$$

where **x** is the original signal and **xˆ** is its recovered version.

In particular, we try the reconstruction using: (1) basis pursuit on single nodes averaging the quality of reconstruction over all seven different signals; (2) the DCS-SOMP algorithm considering a JSM-2 model for the signal ensemble; (3) joint-sparse basis pursuit model (JS-BP) solved with the YALL1 [38] MATLAB package; and (4) gradient projection-based sparse reconstruction (GPSR).

While the BP does not exploit any correlation or *a priori* information and DCS-SOMP and JS-BP try to exploit inter-correlations existing among the different nodes, the GPSR algorithm is well suited, both for periodic and correlated signals, since it presents a weighting factor that can be used to give to the reconstruction algorithms some hints about reconstruction.

With the same nomenclature as in the previous section, the problem of signal reconstruction for GPSR can be expressed as:

(11) $$\mathrm{minimize}\;\left[{\left\|\psi \Phi \alpha -\text{y}\right\|}_{2}^{2}+\tau {\left\|\text{W}\alpha \right\|}_{1}\right]$$

where *τ* is a non-negative parameter providing the relative weight of the ℓ_1_-norm and ℓ_2_-norm in the cost function, while **W** is a diagonal matrix with *ω*_1_,…, *ω_n_* on the diagonal and:

(12) $$\omega_{i}=\frac{1}{\left|\eta_{i}\right|+\epsilon }$$

where *ϵ* > 0 is in order to provide stability, and in general, the weights *η_i_* are free parameters in the convex relaxation whose values could improve the signal reconstruction. The matrix **W** can be in fact used to incorporate *a priori* information about sparsity and can be estimated on-line from inter-or intra-correlation data between sensors and nodes.

In this section, we use the data for the same sensor the day before those involved in the reconstruction as training information for each sensor to obtain the **W** matrix, exploiting the temporal intra-correlation of each node.

In the simulations, the acquisition period before sending out the compressed data toward the base station is two days (more precisely 42 h). During this period, each sensor of each node is sampled, and *M* samples are gathered by the node according to the generated **Φ** matrix. The minimum wake-up time (the minimum inter-sample period) is 5 min, so a maximum number of *N*_acc_ = 512 samples can be gathered by each node for each sensor in one acquisition period. The sparsifying matrix **ψ** is a DCTmatrix that is already demonstrated to be a good basis for compressible natural signals, as highlighted in [31,39]. Each simulation cycle is performed for 100 trials, and for each run, both the measurement matrix and the sampling pattern for the non-uniform random sampling are randomly generated.

**Figure 6:**
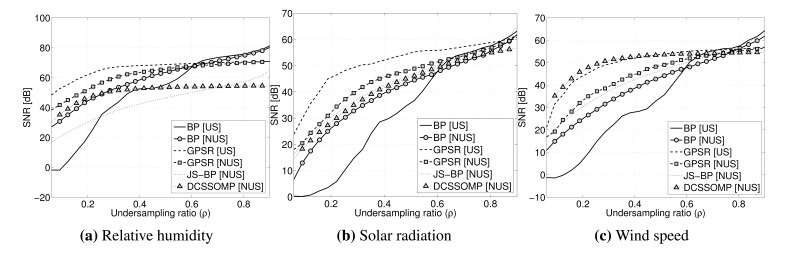
Quality of reconstruction *vs.* the under-sampling ratio for the three kinds of signals taken into consideration. Each signal is reconstructed using all of the algorithms investigated in the paper, varying also the under-sampling pattern.

In Figure 6, the reconstruction quality for each kind of signal averaged over all seven nodes is reported. The plot is done against the under-sampling ratio *ρ* = *M*/*N* defined as the fraction of the samples actually taken with respect to the number of total samples.

The results clearly show how BP does not perform well for all three signals when low under-sampling ratios are considered, achieving an SNR that is lower than the one obtained with all of the other algorithms. Algorithms involving the exploitation of spatial inter-correlation between nodes or temporal intra-correlation achieve a much better reconstruction quality for all of the signals considered. In general, the results show that better reconstruction quality is obtained using the GPSR algorithm. This much higher SNR for reconstruction using GPSR is obtained by giving the reconstruction algorithm useful hints about the signal to reconstruct, as seen in Equation (11). For the wind speed the reconstruction quality guaranteed by GPSR is comparable to that achieved by DCS-SOMP; this is due to the fact that the wind speed, among all of the signals, presents a lower temporal correlation.

The plot also shows that, while for GPSR, the uniform sampling (US) outperforms the non-uniform sampling pattern (NUS), for BP, this is the opposite.

### 6.1. Training Data for GPSR

From the results collected, it follows that the best algorithm able to provide a good reconstruction of the signals is GPSR. In this section, we want to investigate how the training data (the parameters under the form of the **W** matrix in Equation (11)) can influence the reconstruction. This is particularly significant in WSNs where spatial and temporal correlations do exist between different nodes and within the node itself.

In our simulations, we investigate four different scenarios, each aimed to exploit spatial correlation among nodes or temporal correlation within the sensor of interest to create a suitable data training for the GPSR reconstruction.

**Figure 7:**
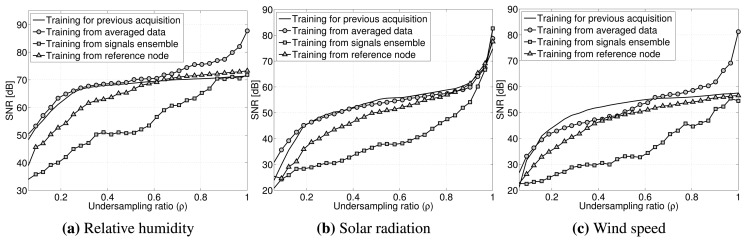
Quality of the reconstruction varying the training data used in the gradient projection-based sparse reconstruction (GPSR) algorithm.

As seen in Figure 7, our training data are obtained: (1) exploiting temporal correlation by using data of the same sensor on the same node reconstructed in the previous acquisition cycle; (2) by averaging a maximum of 10 signals reconstructed in the previous acquisition cycles; (3) by using a pseudo-signal obtained combining the raw data gathered by neighbor nodes; and (4) by using a line-powered node taken as the reference providing uncompressed data placed near the compressing node. This last point is a fictitious case taken as the reference, since it is not always possible to have a line-powered node providing a continuous stream of data, but it is useful to evaluate the recovery when spatially-correlated data are used for reconstruction.

The first result inferred from the simulations output is that, exploiting the spatial correlation using as training for the algorithm the pseudo-signal is not convenient, since the quality of the reconstruction is lower than the one obtained using the other methods.

In general, a better recovery is achieved when data temporally correlated with the signal that we want to recover are used as training data. This is particularly true for periodic signals, such as the environmental signals of interest. The best results in the compression range of interest are obtained by using as training for the GPSR algorithm data coming from the same sensor and node, but gathered in a previous acquisition cycle. This can guarantee the maximum temporal (and obviously, spatial) correlation, giving helpful hints to the reconstruction algorithm to correctly recover the signal.

### 6.2. Energetically Optimal Reconstruction

**Figure 8:**
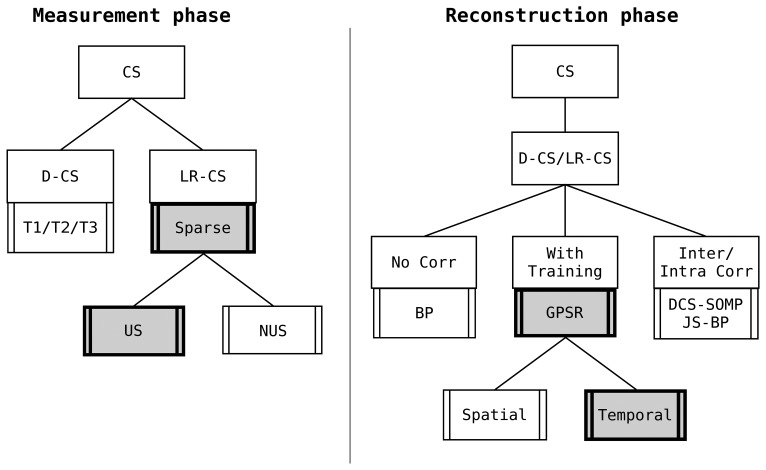
With the same nomenclature previously introduced, this plot highlights the different choices for the measurement and reconstruction phase that permit one to achieve better reconstruction with the minimum energy expenditure.

In Sections 4 and 5, we have investigated the compression phase, coming to the conclusion that a sparse measurement matrix is the best compression matrix to save energy in compression. Afterwards, in Section 6, we have obtained that, among several reconstruction algorithms and using this sparse measurement matrix, GPSR is the best algorithm capable of guaranteeing the higher reconstruction quality. In Figure 8, a graphical review of the best choices in the measurement and reconstruction phase is reported.

Since we have investigated both the power consumption in compression and the reconstruction quality using GPSR, it is possible to address the problem to find the optimal compression parameters able to guarantee good reconstruction quality with the minimum energy expenditure.

**Figure 9:**
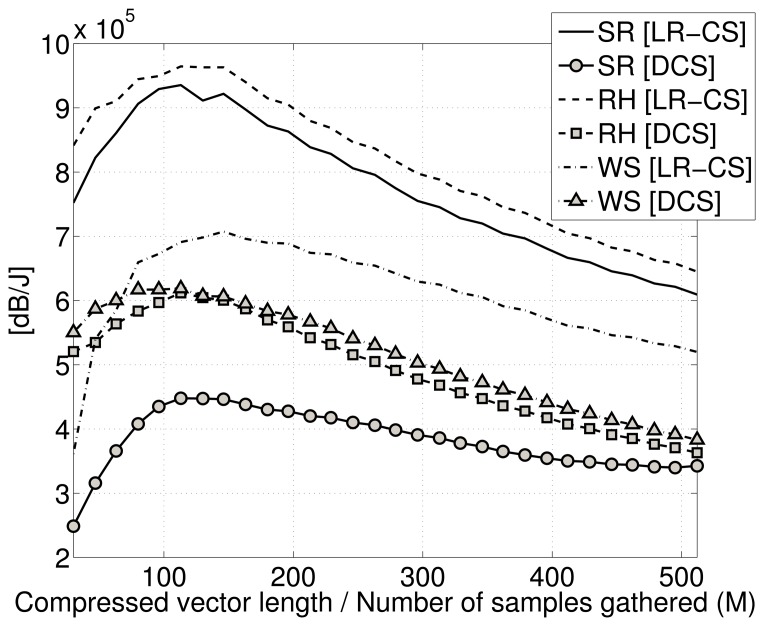
Trade-off between reconstruction and energy consumption for compression. (SR: solar radiation; RH: relative humidity; WS: wind speed; LR-CS: low-rate CS; DCS: digital CS.)

In Figure 9, the trade-off between quality of signal recovery and power consumption is reported, plotting the ratio between quality of reconstruction and the energy spent in compression varying, the under-sampling ratio ρ for low-rate CS and the compression vector size *M* for digital CS.

Looking at the plot, we can see how the curves for LR-CS are always above the curves for the digital CS, meaning that for LR-CS, the compression is energetically cheaper. More precisely, this means that each dB in reconstruction is obtained using less Joules of energy during the compression phase.

Moreover, within the same class of curves, we have a range of compression values *M* and ρ (between *M* = 100 and *M* = 200 for digital CS and ρ = 0.2 and ρ = 0.4 for the low-rate CS) for which the curves present a maximum, identifying the best trade-off between reconstruction quality and power consumption for compression.

Comparing these values with the plots in Figure 6, we can see how in this range, the quality of reconstruction is always > 30 dB, which is a very good reconstruction quality for our goals.

Thus, the low-rate CS with an under-sampling ratio 0.2 ≤ ρ ≤ 0.4 when reconstruction is performed using GPSR with temporally correlated data as training data is able to guarantee an optimal reconstruction > 30 dB with minimum energy used for compression.

## 7. Conclusions

In this paper, we have investigated the application of CS with real COTS hardware, and using an energy consumption model, we have evaluated the impact of different kinds of measurement matrices on the power consumption. We have verified that huge differences do exist according to the compression matrix used and that it is not always convenient to compress data with CS when expensive matrices are used in compression.

Even though low-rate CS seems an optimal solution to save energy, different algorithms for reconstruction exist that do not always guarantee the same recovery quality. Several of these algorithms have been compared against a set of sub-Nyquist-sampled signals taken from a real dataset. Among all of the algorithms considered (each exploiting a different kind of correlation among different nodes within the node itself), GPSR has resulted in being the best algorithm for data recovery when temporally-correlated signals are used as training data.

Finally, an optimal under-sampling ratio and reconstruction algorithm have been identified to be capable of achieving the best reconstruction at the minimum energy cost for the compression.

For future work, we want to explore the possibility to extend low-rate CS to perform in-network compression using distributed scalable algorithms for data gathering and reconstruction, moving from a star network to more complex mesh networks.
